# Calmodulin 1 Regulates Senescence and ABA Response in *Arabidopsis*

**DOI:** 10.3389/fpls.2018.00803

**Published:** 2018-07-02

**Authors:** Cheng Dai, Yuree Lee, In C. Lee, Hong G. Nam, June M. Kwak

**Affiliations:** ^1^College of Plant Science and Technology, Huazhong Agricultural University, Wuhan, China; ^2^Center for Plant Aging Research, Institute for Basic Science, Daegu, South Korea; ^3^Department of New Biology, Daegu Gyeongbuk Institute of Science and Technology, Daegu, South Korea

**Keywords:** calmodulin 1, NADPH oxidase, reactive oxygen species, RPK1, senescence

## Abstract

Cellular calcium acts as a second messenger and regulates diverse developmental events and stress responses. Cytosolic calcium has long been considered as an important regulator of senescence, however, the role of Ca^2+^ in plant senescence has remained elusive. Here we show that the *Calmodulin 1* (*CaM1*) gene, which encodes Ca^2+^-binding protein calmodulin 1, positively regulates leaf senescence in *Arabidopsis*. Yellowing of leaves, accumulation of reactive oxygen species (ROS), and expression of the *senescence-associated gene 12* (*SAG12*) were significantly enhanced in *CaM1* overexpression plants. In contrast, abscisic acid (ABA)-triggered ROS production and stomatal closure were reduced in *amiRNA-CaM1* plants. We found a positive-feedback regulation loop among three signaling components, CaM1, RPK1, and RbohF, which physically associate with each other. RPK1 positively regulates the expression of the *CaM1* gene, and the CaM1 protein, in turn, up-regulates *RbohF* gene expression. Interestingly, the expression of *CaM1* was down-regulated in *rbohD, rbohF*, and *rbohD/F* mutants. We show that CaM1 positively regulates ROS production, leaf senescence, and ABA response in *Arabidopsis*.

## Introduction

Leaf senescence is the terminal stage of leaf development and is genetically programmed. Apparent morphological changes involved in leaf senescence include the yellowing of leaves caused by the degradation of chlorophyll, followed by reduction in photosynthesis and protein synthesis. During senescence, the metabolism and structure of leaf cells continuously change to effectively utilize plant nutrients for the developing parts of the plant, including young leaves, seeds, and fruits (Lim et al., 2007).

Calcium is a universal second messenger that exerts an allosteric effect on many enzymes and proteins in various cellular responses. In plants, calcium signaling is evoked by endogenous and environmental cues, such as drought, salt or osmotic stresses, temperature, light, and plant hormones (Dodd et al., 2010; Steinhorst and Kudla, 2014; Edel and Kudla, 2016; Ranty et al., 2016). Ca^2+^ ions appear to play an important role in plant senescence as well. For instance, exogenously supplied Ca^2+^ delays the senescence of a detached leaf (Poovaiah and Leopold, 1973), and the Ca^2+^ ionophore A23187 rescues MeJA-mediated leaf senescence (Chou and Kao, 1992). Moreover, Ca^2+^-mediated (NO production negatively regulates the expression of senescence-associated genes and production of H_2_O_2_ during the initiation of leaf senescence (Ma et al., 2010).

Calmodulin (CaM), a small Ca^2+^-binding protein, is one of the major calcium sensor proteins conserved in eukaryotes (Chin and Means, 2000; McCormack et al., 2005). Ca^2+^ binding to CaMs causes a conformational change in the protein structure, thereby modifying its interaction with various target proteins, which leads to the transduction of Ca^2+^ signals (Zielinski, 1998, 2002). In *Arabidopsis*, seven genes encode four CaM isoforms: *CaM1/CaM4, CaM2/CaM3/CaM5, CaM6, and CaM7* (McCormack et al., 2005). Four amino acid substitutions differentiate CaM7 from CaM1/CaM4, and one amino acid substitutions differentiate CaM7 from CaM2/CaM3/CaM5, and CaM6. CaM7 is a transcriptional regulator that directly interacts with the promoters of several light-inducible genes, contributing to the regulation of photomorphogenesis (Kushwaha et al., 2008).

Receptor-like Protein Kinase 1 (RPK1) localizes to the plasma membrane and plays an important role in embryo development, plant growth, ABA signaling, and stress responses (Hong et al., 1997; Osakabe et al., 2005, 2010; Nodine et al., 2007; Nodine and Tax, 2008). Additionally, RPK1 positively regulates leaf aging (Lee et al., 2011). The RbohF, an NADPH oxidase, produces reactive oxidation species (ROS) in response to biotic and abiotic stresses in *Arabidopsis* (Torres et al., 2002; Kwak et al., 2003; Joo et al., 2005; Desikan et al., 2006; Zhang et al., 2009). The presence of EF-hand Ca^2+^ binding motifs on NADPH oxidases has suggested a regulatory effect of Ca^2+^ on the enzymatic activity (Torres et al., 1998, 2002), which was further supported by the finding that Ca^2+^ and phosphorylation synergistically activate RbohD NADPH oxidase (Ogasawara et al., 2008). Furthermore, it has been recently shown that RbohF is responsible for RPK1-mediated ROS production, senescence-associated gene expression, and age-induced cell death (Koo et al., 2017).

Several lines of evidence have suggested that cytosolic Ca^2+^ acts as a regulator of senescence in plant, but how senescence is regulated by cytosolic Ca^2+^ remains elusive. Here, we report that *Arabidopsis* CaM1 plays a positive role in RPK1-mediated leaf senescence and ABA response. Senescence phenotypes, including leaf yellowing, ROS accumulation, and expression of the *SAG12* were drastically promoted in *CaM1*-overexpressing plants, whereas plants expressing *amiRNA-CaM1* showed no visible senescence phenotype, which is likely to be due to functional redundancy in the calmodulin gene family. Expression analyses revealed that *CaM1* is positively regulated by RPK1, *RbohF* by CaM1, and *CaM1* by RbohD and RbohF, suggesting a positive-feedback loop among the three signaling components at the transcriptional level.

## Results

### *CaM1* Gene Expression Is Associated With Aging

**FIGURE 1:**
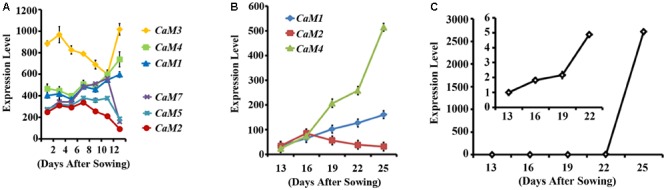
Expression profiles of *CaM* family members in leaves during senescence. **(A)** Expression of the *CaM* genes in rosette leaves, including leaves undergoing senescence 35 days after sowing. Data corresponding to three arrays for each data point were obtained from Genevestigator (https://genevestigator.com/gv/). **(B)** qRT-PCR data showing the expression of *CaM1, CaM2*, and *CaM4* genes in the fourth rosette leaves at the indicated time points. **(C)** qRT-PCR analysis of *SAG12* expression in the fourth rosette leaves of WT plants. The inset shows *SAG12* expression until 22 days after sowing (DAS). Expression levels of genes were normalized to *Actin2*. Error bars represent means ± SEM of three independent experiments.

To identify calmodulin genes that are involved in plant senescence, *in silico* analysis using publicly available microarray data was conducted and showed that the expression of *Arabidopsis CaM* genes can be divided into two groups during leaf senescence: age-dependent increase (*CaM1, CaM3*, and *CaM4*) and age-dependent decrease (*CaM2, CaM5*, and *CaM7*) (no data was available for *CaM6*; **Figure 1A**) (Schmid et al., 2005). This implies that *CaM1, CaM3*, and *CaM4* probably play a positive role in **Arabidopsis** leaf senescence. To verify the microarray data, we examined age dependent- expression pattern of the *CaM* genes: The fourth rosette leaves of *Arabidopsis* plants were collected every 3 days starting with 13-day-old plants and transcript levels of the *CaM1, CaM2*, and *CaM4* genes were analyzed using quantitative RT-PCR (**Figure 1B**). We found that *CaM1* and *CaM4* were indeed up-regulated in the older leaves compared with the younger leaves. The expression pattern was similar to that of the leaf senescence marker, *SAG12* whose expression gradually increased from 13 days after sowing in an age-dependent manner (**Figure 1C**) (Woo et al., 2001). By contrast, the expression of *CaM2* was down-regulated (**Figure 1B**).

**FIGURE 2:**
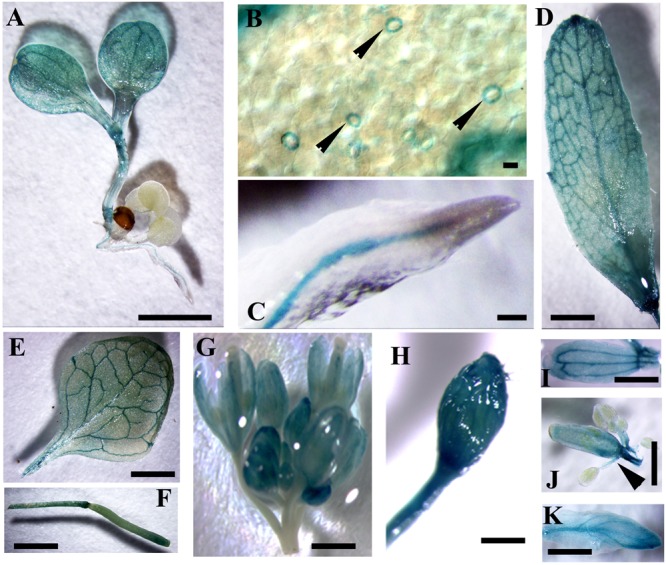
Visualization of *CaM1* expression in *Arabidopsis* transgenic plants harboring a *CaM1pro-GUS* construct. Histochemical GUS staining images of seedlings **(A,B)**, roots **(C)**, cauline leaves **(D)**, rosette leaves **(E)**, silliques **(F)**, flowers **(G,H)**, petals **(I)**, anthers **(J)**, and sepals **(K)** are shown. Arrowheads indicate GUS staining in the guard cells of leaf **(B)** and in filaments **(J)**. Scale bar, 1 cm.

*In silico* analysis showed that the expression level of *CaM1* was similar to that of *CaM4* in most of the tissues, except for reproductive organs (**Supplementary Figures S1A,B**), which was confirmed by qRT-PCR analyses (**Supplementary Figure S1C**). *CaM1* is highly expressed in pollen whereas *CaM4* is highly expressed in dry seeds (**Supplementary Figures S1A,B**). To verify the expression pattern of *CaM1*, at least 10 independent *pCaM1::GUS* transgenic lines were subjected to GUS histochemical analysis. Data revealed that *CaM1* was highly expressed in vascular bundles of cotyledons, roots, and both rosette and cauline leaves (**Figures 2A–E**). *CaM1* was also detected in reproductive tissues, including flowers and siliques (**Figures 2F–K**). Dissection of stained flowers revealed that *CaM1* was expressed in sepals, petals, carpels and filaments, but not in anthers (**Figures 2I–K**). Interestingly, *CaM1* was highly expressed in guard cells (**Figure 2B**), suggesting a role of *CaM1* in stomatal development and/or movement.

### Leaf Senescence Is Accelerated in *CaM1*-Overexpressing Plants

**FIGURE 3:**
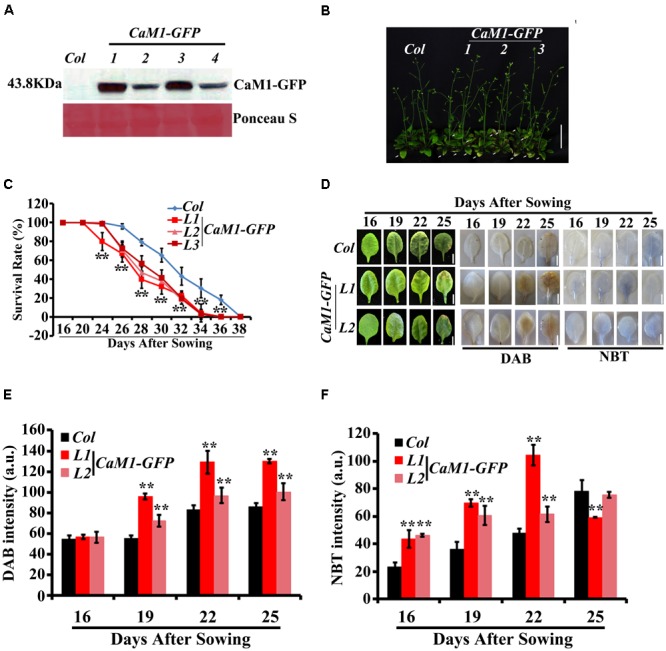
Age-dependent leaf senescence is accelerated in *35S::CaM1-GFP* transgenic lines. **(A)** Detection of CaM1-GFP fusion protein in four independent transgenic lines using Western blot analysis with anti-GFP antibody. Approximately 10-μg protein of each sample was loaded on the gel and stained with Ponceau S. **(B)** Yellowing phenotypes of rosette leaves of *35S::CaM1-GFP* transgenic lines. The picture was taken 31 days after sowing. Arrows indicate leaf yellowing. **(C)** Survival rates of WT and *CaM1-GFP*. Plants with third and fourth rosette leaves showing yellowing were counted. Data are from two independent experiments. For each experiment, 20 plants were analyzed. **(D–F)** Analysis of superoxide and H_2_O_2_ levels using NBT and DAB, respectively, in the fourth rosette leaves of WT and *35S::CaM1-GFP* transgenic lines L1 and L2 at the indicated time points **(D)**. Quantitative analysis of NBT **(E)** and DAB **(F)**. Error bars represent means ± SEM of three independent experiments. Statistical analysis was performed using heteroscedastic *t*-test (^∗^*p* < 0.05; ^∗∗^*p* < 0.01). Scale bar, 1 cm.

Previously, we have shown that CaM4 plays a role in RPK1-mediated leaf senescence in *Arabidopsis* (Koo et al., 2017). Thus, CaM1 was chosen to investigate its role in plant senescence. As a first step, transgenic plants expressing *35S::CaM1-GFP* were generated. Western blot analyses using anti-GFP antibody showed enhanced accumulation of CaM1 in the transgenic plants (**Figure 3A**). Leaves of three independent *35S::CaM1-GFP* transgenic lines turned yellow earlier than the WT (**Figures 3B,C**). No other growth abnormalities including seed size and mass were observed in the *35S::CaM1-GFP* transgenic lines compared with the WT (**Supplementary Figure S2**). It is known that the production of ROS is induced during senescence in both animals and plants (Lim et al., 2007; Vigneron and Vousden, 2010). Thus, we monitored the accumulation of H_2_O_2_ and superoxide, respectively in the leaves of WT and *35S::CaM1-GFP* transgenic lines at different developmental stages using DAB and NBT staining. We found that ROS accumulation was detected earlier in the transgenic plants than in WT (**Figures 3D–F**). Higher accumulation of H_2_O_2_ and superoxide was detected at 16 and 19 days, respectively, in the transgenic plant leaves after sowing (**Figures 3E,F**).

**FIGURE 4:**
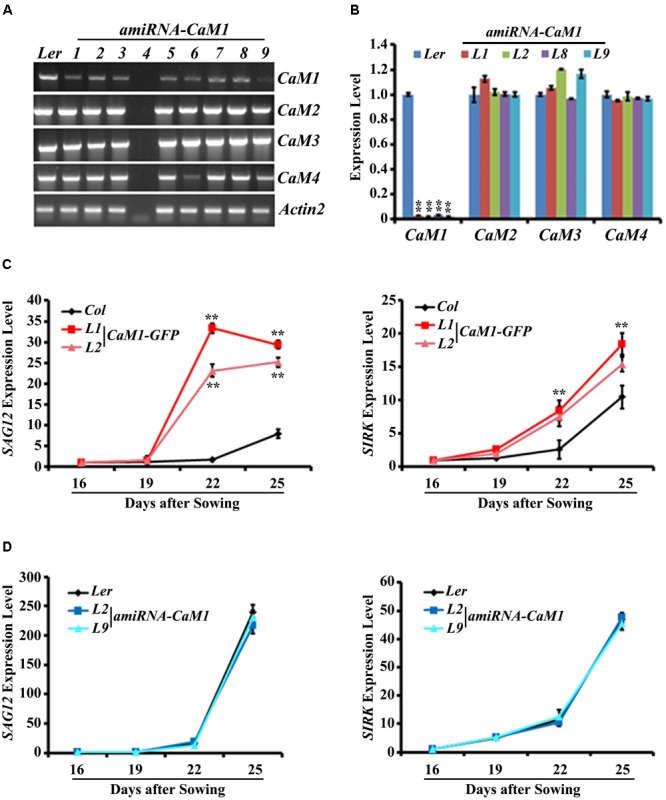
The expression of senescence marker genes is accelerated in the *35S::CaM1-GFP* transgenic lines. **(A,B)** Expression analysis of *CaM* genes in T3 homozygous *35S::amiRNA-CaM1* lines by RT-PCR **(A)** and qRT-PCR **(B)**. **(C,D)** qRT-PCR analysis of senescence-related genes in *35S::CaM1-GFP* transgenic lines **(C)** and *35S::amiRNA-CaM1* plants **(D)**. Expression levels of the genes were normalized to *Actin2*. Error bars represent means ± SEM of three independent experiments. Statistical analysis was performed using heteroscedastic *t*-test (^∗∗^*p* < 0.01).

To further examine the role of *CaM1* in leaf senescence, we generated transgenic plants in which the expression of *CaM1* was knocked down using an artificial microRNA targeting *CaM1* (*amiRNA-CaM1*). In the *amiRNA-CaM1* transgenic lines, the transcript levels of *CaM2, CaM3*, and *CaM4* were not significantly changed, whereas *CaM1* transcript level was significantly down-regulated compared with the WT (**Figures 4A,B**). This result implies that the *amiRNA-CaM1* specifically down-regulates *CaM1* transcripts, allowing us to examine cellular responses that CaM1 particularly mediates.

qRT-PCR analyses showed that early leaf senescence phenotype of the *35S::CaM1-GFP* transgenic lines was strongly correlated with the drastically enhanced expression of senescence markers *SAG12, SIRK, ATG2, and ATG5* compared with the WT (**Figure 4C** and **Supplementary Figure S3A**). This suggests that *CaM1* overexpression results in various senescence-associated changes, including yellowing of leaves, enhanced production of H_2_O_2_ and superoxide, and up-regulation of the senescence marker genes. In contrast, no altered senescence phenotypes were observed in the *amiRNA-CaM1* transgenic lines, and the expression level of *SAG12, SIRK, ATG2, and ATG5* in the *amiRNA-CaM1* transgenic lines was comparable with that of the WT (**Figure 4D** and **Supplementary Figure S3B**). *cam4* knockout mutants displayed no altered senescence phenotype (**Supplementary Figures S3C,D**). This result suggests that there is functional redundancy between *CaM1* and *CaM4* and that CaM1/CaM4 functions as a positive regulator of age-dependent leaf senescence.

### CaM1 Positively Regulates ABA-Induced ROS Production

**FIGURE 5:**
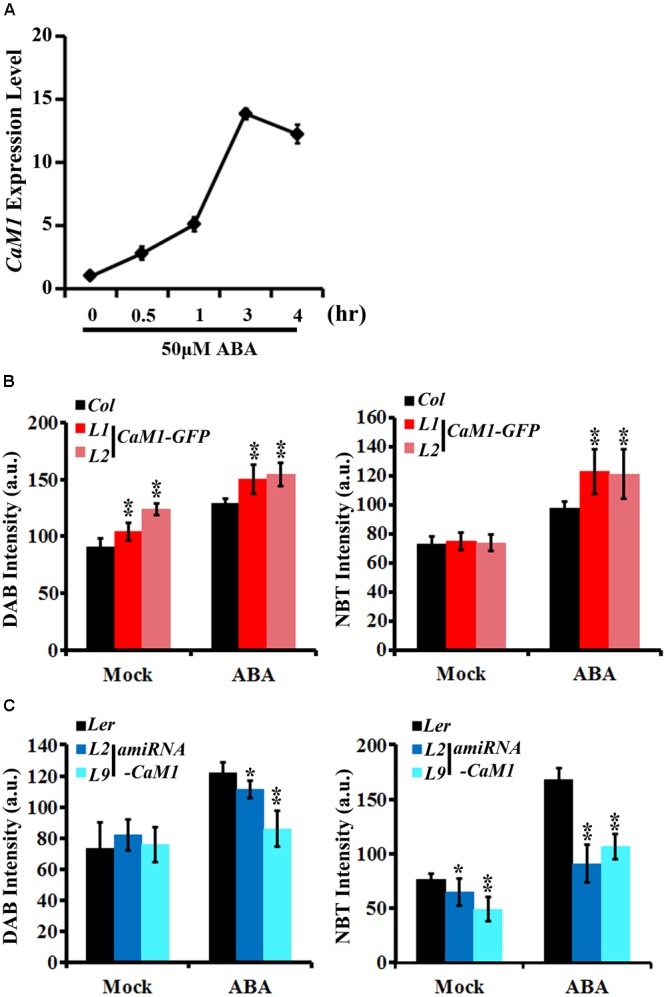
CaM1 positively regulates ABA-induced ROS production. **(A)** qRT-PCR analysis reveals that the expression of *CaM1* is induced 0.5 h after ABA treatment (50 μM). *Actin*2 was used as an internal control. **(B,C)** Quantitative analyses of ROS staining in the fourth rosette leaves of 3-week-old plants expressing *35S::CaM1-GFP* **(B)** and *35S::amiRNA-CaM1* **(C)**. Leaves were treated with 100 μM ABA for 1 h and stained with DAB and NBT to visualize accumulation of H_2_O_2_ and superoxide, respectively. Leaves without ABA treatment (mock) were used as a control. Error bars represent means ± SEM of two independent experiments. Each data point represents 10 leaves. Statistical analysis was performed using heteroscedastic *t*-test (^∗^*p* < 0.05; ^∗∗^*p* < 0.01).

We found that the production of H_2_O_2_ and superoxide was increased in the *35S::CaM1-GFP* transgenic plants during leaf senescence (**Figure 3C**). Since ABA is also known to induce ROS production (Pei et al., 2000; Kwak et al., 2003) as well as leaf senescence (Zhao et al., 2016), we examined whether *CaM1* is linked to ABA-mediated ROS production. The expression of *CaM1* was induced within 30 min of ABA treatment, reaching a peak at 3 h (**Figure 5A**). We also determined ROS levels in leaves of 3-week-old plants before and after the ABA treatment using DAB and NBT staining. The steady-state H_2_O_2_ level in *35S::CaM1-GFP* plants was higher than in WT plants prior to the ABA treatment (**Figure 5B**). Although ABA induced H_2_O_2_ and superoxide production in both WT and the *35S::CaM1-GFP* transgenic plants, we found a higher production of H_2_O_2_ and superoxide in the *35S::CaM1-GFP* transgenic plants (**Figure 5B**). By contrast, ABA-induced ROS production was reduced in *amiRNA-CaM1* transgenic lines (**Figure 5C**). These results imply that CaM1 positively regulates ABA-induced ROS production.

**FIGURE 6:**
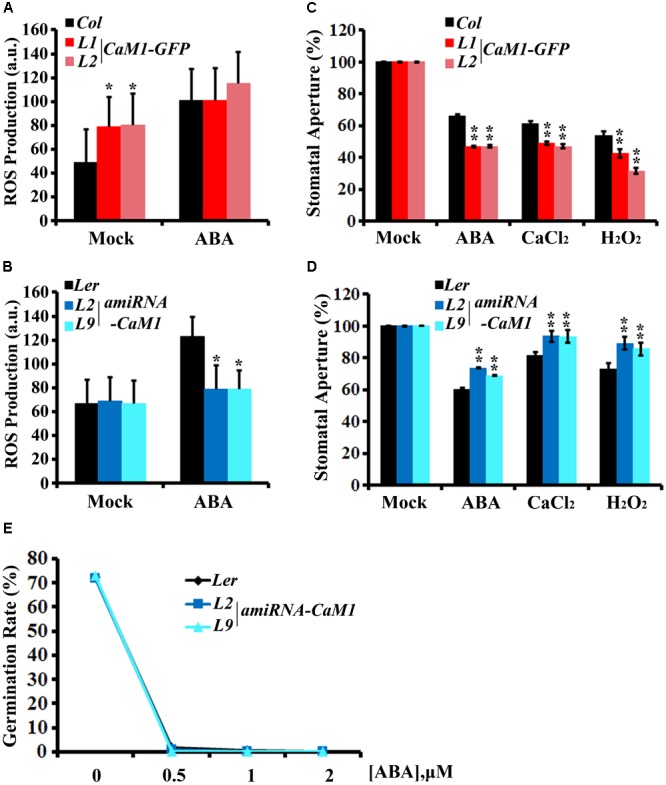
CaM1 mediates ABA-induced ROS production in guard cells and stomatal closure. **(A,B)** ABA-induced ROS accumulation in guard cells of *35S::CaM1-GFP* plants **(A)** and of the *35S::amiRNA-CaM1* transgenic plants **(B)**. ROS levels were measured 30 min after ABA treatment (50 μM) using 2′7′-dichlorofluorescein diacetate. Error bars represent means ± SEM of three independent experiments (*n* = 50). **(C,D)** ABA (5 μM)-, CaCl_2_ (2 mM)-, and H_2_O_2_ (100 μM)-induced stomatal closure in *35S::CaM1-GFP* plants **(C)** and *amiRNA-CaM1* plants **(D)** (*n* = 100). **(E)** Germination rate of WT (*Ler*) and transgenic *amiRNA-CaM1* L2 and L9 seeds were measured after 7 days of incubation on MS medium containing 0, 0.5, 1, or 2 μM ABA without sucrose. Error bars represent means ± SEM of three independent experiments. Statistical analysis was performed using heteroscedastic *t*-test (^∗^*p* < 0.05; ^∗∗^*p* < 0.01).

Stomatal aperture is one of the targets of ABA-mediated signaling in plants. Furthermore, ROS have been shown to act as a positive regulator of ABA signaling in guard cells (Pei et al., 2000; Kwak et al., 2003). Thus, we investigated whether CaM1 functions in guard cell ABA response. We determined ROS levels in guard cells of the fourth rosette leaves harvested from 25-day-old plants before and after ABA treatment. H_2_-DCFDA mainly detects H_2_O_2_ and can be easily quantitated in guard cells (Kwak et al., 2003). The steady-state ROS level in *35S::CaM1-GFP* guard cells was higher than in WT guard cells (**Figure 6A**). Although no significant differences were detected between WT and the *35S::CaM1-GFP* transgenic lines after ABA treatment (**Figure 6A**), it is likely to be due to the saturation of DCF fluorescence in the cells analyzed. By contrast, ABA failed to trigger ROS production in guard cells of *amiRNA-CaM1* transgenic plants (**Figure 6B**). Overexpression of *CaM1* enhanced ABA-induced stomatal closure, whereas down-regulation of *CaM1* inhibited ABA-induced stomatal closure (**Figures 6C,D**). These results imply that CaM1 positively regulates ABA-induced ROS production in guard cells, thereby modulating stomatal movements.

Calcium and H_2_O_2_ are second messengers that induce stomatal closure (Pei et al., 2000; Zhang et al., 2001; Kwak et al., 2003). In the *CaM1* overexpression plants, calcium- and H_2_O_2_-induced stomatal closure was enhanced (**Figure 6C**). In contrast, down-regulation of *CaM1* reduced the calcium- and H_2_O_2_-induced stomatal closure (**Figure 6D**), implying that CaM1 functions in calcium- and H_2_O_2_-mediated stomatal closure. More importantly, even though there is functional redundancy between *CaM1* and *CaM4* in leaf senescence (**Figure 4** and **Supplementary Figure S3**) and ABA-mediated seed germination (**Figure 6E**), *CaM1* appears to play a unique role in the regulation of ROS production in guard cells.

### Positive-Feedback Regulation Among *RPK1, CaM1*, and *RbohF*

**FIGURE 7:**
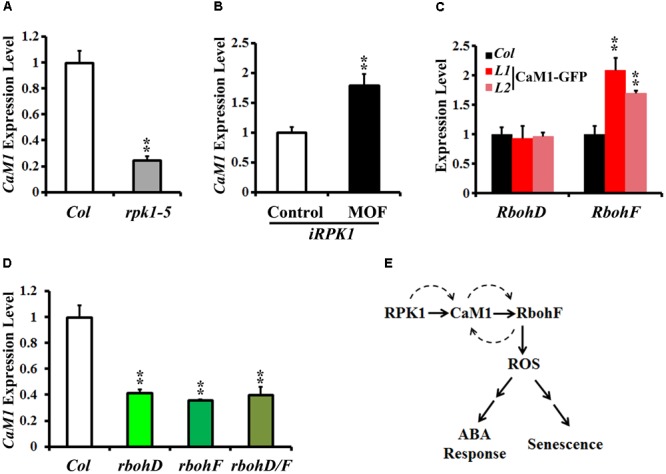
RPK1 positively regulates the expression of CaM1, which in turn activates a positive-feedback loop between CaM1 and RbohD/F. **(A,B)** qRT-PCR analysis of *CaM1* expression in *rpk1-5* mutants **(A)** and *iRPK1* transgenic plants after methoxyfenozide (MOF) treatment **(B)**. **(C)** qRT-PCR analysis of *RbohD* and *RbohF* transcript level in the *35S:CaM1-GFP* transgenic plants. **(D)** qRT-PCR analysis of *CaM1* in *rbohD, rbohF*, and *rbohD/*F mutants. Error bars represent means ± SEM of three independent experiments. **(E)** A working model for the regulation among *RPK1, CaM1*, and *RbohF* genes. Solid arrows indicate cellular events at the protein level, and dashed arrows indicate feed-forward and feedback regulation at the transcription level. Statistical analysis was performed using heteroscedastic *t*-test (^∗∗^*p* < 0.01).

Peptide sequence alignments of CaM proteins revealed a high level of sequence similarity (**Supplementary Figure S4**). Phylogenetic analysis indicated that CaM1 is closest to CaM4 (**Supplementary Figure S5**). Although the sequence similarity between *CaM1* and *CaM4* coding sequences was 89.56% at the nucleotide level, the amino acid sequences encoded by these genes were identical (**Supplementary Figure S4**). We have previously shown that CaM4 physically interacts with RPK1 and RbohF (Koo et al., 2017). To test whether CaM1 also acts with RPK1 and RbohF and to investigate potential regulation among *RPK1, CaM1*, and *RbohF* at the transcriptional level, we examined the expression of *CaM1* in *rpk1-5* null mutants and *iRPK1* transgenic plants in which the expression of *RPK1* is under the control of ecdysone-inducible promoter (Lee et al., 2011). In *rpk1-5*, the expression of *CaM1* was significantly down-regulated in leaves of 25-day-old plants (**Figure 7A**; *p* < 0.01). By contrast, *RPK1* expression was notably up-regulated in the *iRPK1* plants when treated with methoxyfenozide, the chemical inducer of the promoter (Padidam, 2003) (**Figure 7B**). These results indicate that the expression of *CaM1* is positively regulated by *RPK1*. However, no significant changes in the expression of *RPK1* were detected in the *35S::CaM1-GFP* or *35S::amiRNA-CaM1* transgenic lines (data not shown), suggesting that *RPK1* is not transcriptionally regulated by *CaM1*.

In addition, we found that the expression of *RbohF* was up-regulated in the 25-day-old *35S::CaM1-GFP* transgenic plants, whereas the expression of *RbohD* was not altered (**Figure 7C**). Interestingly, the expression of *CaM1* was down-regulated in *rbohD, rbohF*, and *rbohD/F* double mutants (**Figure 7D**). Taken together, these data suggest a positive-feedback loop among the *CaM1, RbohF*, and *RPK1* genes at the transcriptional level (**Figure 7E**).

## Materials and Methods

### Plant Materials and Growth Conditions

T-DNA insertional line GK-309E09 (*cam4, AT1G66410*) was obtained from the Arabidopsis Biological Resource Center. Seeds of wild type (WT) *Arabidopsis, cam4*, and *35S::CaM1-GFP* (Columbia background) and *35S::amiRNA-CaM1* (Landsberg background) transgenic lines were germinated in soil. Seedlings were grown in the growth chamber at 21°C ± 1°C under 16 h light/8 h dark photoperiod. Plants at the 3–5-week-old stage were used for various experiments described below.

### Dichlorofluorescein Diacetate (H_2_DCF-DA) Assay of Guard Cells

The production of H_2_O_2_ in guard cells was examined using 2′7′-(H_2_DCF-DA, Molecular Probes, Eugene, OR, United States) as described previously (Murata et al., 2001) with slight modifications. Epidermal strips were prepared from 4 to 5-week-old WT, and transgenic *35S::CaM1-GFP* and *35S::amiRNA-CaM1* plants using a blender and incubated in buffer (5 mM KCl, 10 mM MES–Tris, pH 6.15) under white light (95 E m^-2^ s^-1^) for 2 h. Subsequently, H_2_DCF-DA (50 μM) was added to the solution containing the epidermal strips and the mixture was incubated on an orbital shaker at 70 rpm for 30 min. Finally, 50 μM ABA was added to the buffer and incubated for 10 min. Images of guard cells were taken under UV light (one 2-second UV exposure per sample) using a fluorescence microscope equipped with a digital camera (Axiovert 200, Zeiss) (Murata et al., 2001). The fluorescence intensity of guard cells was measured using Image J.

### Constructs and Plant Transformation

Artificial microRNA of *CaM1* (*amiRNA-CaM1*) was designed by WMD3^3^. Primers, including attb1-oligo A, attb2-oligo B, CaM1-I-miR-s, CaM1-II-miR-a, CaM1-III-miR^∗^s, and CaM1-IV miR^∗^a were used to PCR amplify on pRS300 (Schwab et al., 2006). The PCR product, *amiRNA-CaM1* was first cloned into the Entry vector pDONR^TM^/Zeo (12535-035, Invitrogen) and then into the pMDC32 vector using Gateway LR cloning (Gateway^®^ LR Clonase^®^ II Enzyme mix, 11791-100, Invitrogen) (Curtis and Grossniklaus, 2003). The promoter region of *CaM1* was amplified by pair of primers CaM1p-attB1 and CaM1p-attB2, and the coding region of *CaM1* was amplified by pair of primers CaM1-attB1 and CaM1-attB2. The PCR products were cloned into the Entry vector pDONR^TM^/Zeo and then into the pMDC162 vector for *pCaM1::GUS* and pMDC43 for *35S::CaM1-GFP* (Schwab et al., 2006). These constructs were introduced into WT *Arabidopsis* using *Agrobacterium*-mediated transformation via the floral dip method (Clough and Bent, 1998). The primer sequences used for plasmid construction are provided in **Table 1**.

**Table 1:** The primer sequences used for plasmid construction.

Primer Name	DNA Sequence (5′-3′)
CaM1-I miR-s	gaTTGAACGCATAACCGTTCCTAtctctcttttgtattcc
CaM1-II miR-a	gaTAGGAACGGTTATGCGTTCAAtcaaagagaatcaatga
CaM1-III miR^∗^s	gaTAAGAACGGTTATCCGTTCATtcacaggtcgtgatatg
CaM1-IV miR^∗^a	gaATGAACGGATAACCGTTCTTAtctacatatatattcct
CaM1-attB1	GGGGACAAGTTTGTACAAAAAAGCAGGCTGCATGGCGG ATCAACTCACTGACGAA
CaM1-attB2	GGGGACCACTTTGTACAAGAAAGCTGGGTCCTTAGC CATCATAATCTTGAC
CaM1p-attB1	GGGGACAAGTTTGTACAAAAAAGCAGGCTGCCGAGGTA TCTTTTAGATAT
CaM1p-attB2	GGGGACCACTTTGTACAAGAAAGCTGGGTCAGCTTCTTC GAGAAATCGTC

### RT-PCR Analysis

Total RNA was extracted from WT and transgenic lines using the TRIzol reagent (15596-026, Invitrogen) and subsequently used for cDNA synthesis using RevertAid Reverse Transcriptase kit (#EP0441, Thermo). The primer pairs CaM1-qs and CaM2-qa, CaM2-qs and CaM1-qa, CaM4-qs/-qa, RPK1-qs/-qa, SAG12-qs/-qa, SIRK-qs/-qa, ATG2-qs/-qa, ATG5-qs/-qa, RbohD-qs/-qa, and RbohF-qs/-qa were used to examine the expression of *CaM1, CaM2, CaM4, RPK1, SAG12, SIRK, ATG2, ATG5, RbohD*, and *RbohF*, respectively, using qPCR. *Actin2* amplified using the primer pair Actin-1/-2 was used as an internal positive control.

For semi-quantitative RT-PCR, equal amounts of first-strand cDNAs were used as templates for PCR amplification. The primer pairs CaM1-s/-a, CaM2-s/-a, CaM3-s/-a, and CaM4-s/-a were used to analyze the expression pattern of *CaM1, CaM2, CaM3*, and *CaM4*, respectively, in the different *amiRNA-CaM1* transgenic lines. The *Actin2* gene was used as an internal control. The primer sequences used for qRT and RT-PCR analysis are provided in **Table 2**.

**Table 2:** The primer sequences used for qRT and RT-PCR analysis.

Primer Name	DNA Sequence (5′-3′)
RPK1-qs	CGTGGGCTCATATGATGTTG
RPK1-qa	AGAAGGCTGGATTCGTTTCA
RbohD-qs	TCTGCCAAGTTTTGGGAATGCTTAG
RbohD-qr	TTGGCATCAAAAGCTTTCGTCTGAG
RbohF-qs	CCGTTCTGGTTCTTATTCGGTTCG
RbohF-qa	CAGGTGCGGAAGTAATTGAGAATGG
CaM1-qs	TGGGAACAGTTATGCGTTCA
CaM1-qa	AACCGTTCTGGTGTTTGTCG
SAG12-qs	ACGATTTTGGCTGCGAAGG
SAG12-qa	TCAGTTGTCAAGCCGCCAG
SIRK-qs	AAGATGGCGGACTTCGGGTTATCTA
SIRK-qa	GCACCTTCTCTGTTTTTGAGCTTGC
ATG2-qs	AGCCGGGGCTCATGATATTTTATTG
ATG2-qa	TGTGCGGACTAATGCAGAAGCTG
ATG5-qs	GACAGCAAGAATTCCTGTTCGGTTG
ATG5-qa	TTTGCGCTCTGTCTCCCATAAACTC
CaM1-s	GAGAGACGACTCTGAATCCA
CaM1-a	CCAACCCATCGGTTTCAATCC
CaM2-s	ACGAATCGTCTCACAAACTCTTTC
CaM2-a	AAGGAGAAAGCCGAAGAAGTTG
CaM3-s	CGTACCCGATAAATACGGTTG
CaM3-a	ACCTCGAGTCCCATGAATAA
CaM4-s	TAATTGTTTTTGTCGGTCGCTAAG
CaM4-a	TCACCACTTTTATTCTTCATT

### Diaminobenzidine (DAB) and Nitroblue Tetrazolium (NBT) Staining

For all histochemical staining experiments, fourth rosette leaves were detached at different time points (13, 16, 19, 22, and 25 days) and treated with 100 μM ABA, 100 μM H_2_O_2_, or 2 mM CaCl_2_ for one hour. DAB and NBT staining were performed as described (Lee et al., 2011). The leaves were submerged in NBT solution [1 mg/mL (NBT, N5514, Sigma), 10 mM NaN_3_, and 10 mM potassium phosphate, pH 7.8] or DAB solution [1mg/ml 3,3′-DAB, D12384, Sigma), pH 3.8] and stained for 30 min at room temperature. Samples were then boiled in 95% ethanol for 10 min and stored in 60% glycerol. After staining, all samples were mounted on slides and photographed using a digital camera (G12, Canon). To quantify DAB and NBT staining, the stained pixels were obtained from 6 to 12 leaves per genotype using the channels function in Adobe Photoshop and their intensity was measured using the TotalLab100 program (Nonlinear Dynamics).

### Stomata Movement Assay

Stomatal assays were performed as described (Desikan et al., 2002). Briefly, epidermal strips were prepared from 3- to 4-week-old rosette leaves and floated abaxial side down in 10 mM MES buffer (pH 6.15) containing 20 mM KCl for 2.5–3 h under white light (95 E m^-2^ s^-1^) to open the stomata. Following this, 100 μM H_2_O_2_ or 2 mM CaCl_2_ was added to the buffer and incubated for another 1–2 h to assay stomatal closure. Pictures of stomata were taken using a microscope equipped with a digital camera (Axiovert 200, Zeiss). The guard cell aperture was analyzed using Image J.

### Seed Germination Assay

Dry seeds were collected and stored in a dehumidifier cabinet for at least 2 months before testing the germination capacity of seeds. Seeds were surface-sterilized using 20% commercial Clorox bleach for 15 min, and washed five times with sterile water. At least 100 sterilized seeds were plated on MS medium (MSP02-10LT, Caisson) containing 0.8% (w/v) Bacto Agar (Difco, BD) supplemented with different concentrations (0.5, 1, 2 μM) of ABA solution (A4906, Sigma-Aldrich) prepared in methanol. Seeds plated on MS media without ABA were used as control. Seeds were stratified in the dark at 4°C for 3 d and then transferred to the growth chamber maintained at 22°C with a 16 h light/8 h dark photoperiod. Germination was defined as the first sign of emergence of green cotyledons and scored daily for 7 days. The germination results were calculated based on at least three independent experiments.

### Histochemical Staining

Positive transgenic plants were identified using GUS staining as described (Jefferson et al., 1987). Pictures were taken using a digital camera (G12, Canon) or microscope (STEMI SV6, Zeiss) equipped with a digital camera.

### Phylogenetic and Alignment Analysis

Homolog sequences of *Zea mays* (gi|162463080, gi|162463001, gi|162462264, gi| 162464382, gi|226490894, gi|226496461, gi|226507438, gi|226507713, gi| 226958443, and gi|293334895), *Glycine max* (gi|351721559, gi|310563, gi|351721835, gi|351722047, gi|170072, gi|170076, and gi|170074), *Chlamydomonas reinhardtii* (gi|159490918 and gi|158280344), *Acyrthosiphon pisum* (gi|209870032), *Triticum aestivum* (gi|291246022), *Brassica napus* (gi|497992), *B. juncea* (gi|899058), *B. oleracea* (gi|374922809, gi|374922811, gi|374922813, and gi|374922807), *Hevea brasiliensis* (gi|313767030), *Arabidopsis thaliana* (CaM1 to CaM7), *Medicago truncatula* (gi|355509904 and gi|357477127), *Vitis vinifera* (VIT_18s0001g03880.t01) and *Oryza sativa* [LOC_Os05g41200.1 (*OsCaM9*), LOC_Os07g48780 (*OsCaM1-1*), LOC_Os03g20370 (*OsCaM1-2*), LOC_Os01g16240 (*OsCaM1-3*), LOC_Os11g03980 (*OsCLM2*) and gi|17066588] were obtained from NCBI^4^ or ABRC (see footnote 2). Sequence alignments were generated using Clustal Omega^5^. Alignments between different species were adjusted for the construction of phylogenetic tree. Neighbor-joining analyses were performed using MEGA5 (Tamura et al., 2011) with pair-wise deletion, Poisson correction set for the distance model and 1,000 bootstrap replicates.

### Analysis of Seed Yield

Average seed mass was determined by weighing 1,000 dry seeds. The weight of at least five independent batches was measured. To analyze relative seed size, seed images were captured on an Epson V370 scanner with the supplied software, which were then analyzed by SmartGrain software (Tanabata et al., 2012). More than 200 seeds per plant were analyzed.

## Discussion

In this report, we investigated the function of the senescence-induced gene *CaM1*. Overexpression of *CaM1* in WT plants enhanced several aspects of age-dependent senescence, including chlorophyll content, ROS production, and expression of senescence marker genes (**Figures 3, 4**). These results suggest that *CaM1* acts as a positive regulator of age-dependent leaf senescence. Our previous study has shown that Ca^2+^ induces the expression of the senescence marker gene *SIRK* via *CaM4* (Koo et al., 2017). In this study, we found enhanced senescence phenotypes in *CaM1*-overexpressing plants (**Figure 3**). These two studies demonstrate that CaM1/CaM4 together with Ca^2+^ positively regulate age-dependent leaf senescence. On the contrary, calcium signaling plays a negative role in MeJA- and NO-mediated leaf senescence (Chou and Kao, 1992; Ma et al., 2010). Thus, it is plausible that other members of the *CaM* gene family might function in the process of leaf senescence. Differences between the expression profiles of various *CaM* genes and antagonistic functions of calcium signaling in leaf senescence suggest that CaM proteins fine-tune the balance of calcium signaling in leaf senescence.

### CaM1 Functions in RPK1-Mediated Leaf Senescence

Receptor-like potein kinase 1 is a plasma membrane-localized receptor kinase (Osakabe et al., 2005) and plays multiple roles in various cellular signaling and developmental processes, including embryo development (Nodine et al., 2007; Nodine and Tax, 2008), plant growth, stomatal opening, stress response (Hong et al., 1997; Osakabe et al., 2005, 2010), and senescence (Lee et al., 2011; Koo et al., 2017). How RPK1 controls these diverse cellular processes may be partly explained by the proteins interacting with RPK1 in those processes. It has been suggested that calcium and CaM play a role in receptor kinase-mediated cellular processes (Oh et al., 2012). Previous studies have shown that RPK1 interacts and phosphorylates a serine residue on CaM4 (Koo et al., 2017). The expression of *CaM1* and *CaM4* was induced during leaf aging (**Figure 1B**), and knockdown of *CaM1* or *CaM4* did not affect leaf senescence (**Figure 4** and **Supplementary Figure S3**). These data indicate that CaM1 and CaM4 are functionally redundant in RPK1-mediated leaf senescence. Ectopic expression of *RPK1* in *Arabidopsis* at a young developmental stage did not result in senescence (Lee et al., 2011), and *CaM1* overexpression did not result in detectable senescence phenotypes at early developmental stages (16-day-old plants; **Figure 3D**), suggesting that the RPK1–CaM1 module regulates senescence in conjunction with other senescence-related components.

### CaM1 Positively Regulates ROS Production

Cellular ROS homeostasis and signaling are crucial parts of signaling networks in plants (Miller et al., 2009). Several lines of evidence show crosstalk between ROS and calcium signaling. For example, NADPH oxidase RHD2/RbohC produces ROS that stimulate hyperpolarization-activated Ca^2+^ channels, leading to the formation of a tip-focused Ca^2+^ gradient (Foreman et al., 2003; Takeda et al., 2008). Plasma membrane Ca^2+^-permeable channels in guard cells are activated by ABA via ROS (Pei et al., 2000; Murata et al., 2001). Because the *Arabidopsis* genome encodes 10 NADPH oxidase genes that contain the conserved EF-hand Ca^2+^-binding motifs in their extended N-terminal regions, a regulatory effect of Ca^2+^ on the activity of NADPH oxidases in plants is expected (Torres et al., 1998). Here, we present data showing a reduction in ABA-induced ROS production in *amiRNA-CaM1* plants (**Figure 5C**) and enhanced ROS levels in *35S::CaM1-GFP* plants without exogenous ABA treatment (**Figure 5B**), suggesting that CaM1-mediated calcium signaling promotes ABA-mediated ROS production. In addition, our data provide evidence that the expression of *RbohF* is positively regulated by CaM1 (**Figure 7C**) and show a positive-feedback regulation between RbohF and CaM1 (**Figure 7D**). Since RbohD also positively regulates CaM1 expression (**Figure 7D**), it will be of interest to test the specificity of the regulation between calmodulins and NADPH oxidases.

ROS modulate activities of target proteins or expression of genes by changing the redox state in the cell (Schmidt and Schippers, 2015; Saxena et al., 2016). A recent study has shown that the ratio between superoxide and H_2_O_2_ in roots determines the cell fate of either differentiation or proliferation, in which NADPH oxidases appear to function (Tsukagoshi et al., 2010). It would be interesting to test whether the ratio between different kinds of ROS also plays a role in age-dependent cell death in leaves. Furthermore, identification of the direct cellular targets of ROS will further shed light on our understanding of ROS-mediated plant senescence.

### Diversified Functions of CaMs With High Level of Sequence Similarity

Peptide sequence alignments of CaM proteins and phylogenetic analysis revealed a high level of sequence similarity among the family members (**Supplementary Figures S4, S5**). Among the seven genes encoding CaM in *Arabidopsis, CaM7* is the least related genes with others (**Supplementary Figure S4**), but only one and four amino acid substitutions differentiate CaM7 from CaM2/CaM3/CaM5 and CaM1/CaM4, respectively. One possible explanation for the conservation of *Arabidopsis CaM* genes is that CaMs share similar functions. Alternatively, different *CaM* genes might have evolved distinct expression patterns or regulatory behaviors to ensure important cellular functions. Recent evidence supports the latter. An expression analysis using the publicly available data^1^ shows that spatial expression of six *CaM* genes is differently regulated (**Supplementary Figure S1**), and biochemical analysis indicates functional non-redundancy in CaMs (Liao et al., 1996; Reddy et al., 1999; Kohler and Neuhaus, 2000). CaM2, CaM4, and CaM6 activates NAD kinase and phosphodiesterase with different kinetics *in vitro* (Liao et al., 1996; Reddy et al., 1999), and CaM2 and CaM4 bind to cyclic nucleotide-gated ion channels and a kinesin-like motor protein with different affinities (Reddy et al., 1999; Kohler and Neuhaus, 2000). These findings suggest that CaMs may exert different functions through their binding targets that may be located in different cellular compartments.

The expression analysis shows that *CaM* genes encoding identical proteins (CAM2/CAM3/CAM5 and CAM1/CAM4) share a similar expression pattern (**Supplementary Figure S1**), implying functional redundancy in the closest paralogs, as observed in the senescence phenotype of the *amiRNA-CaM1* and *cam4* plants (**Figure 4**). Nevertheless we identified a specific role for *CaM1* in the ABA-mediated ROS production and stomatal closure (**Figures 5, 6**). Fine-tuning of gene expression in a cell type-specific or stimulus-specific manner and/or different post-transcriptional regulation between *CaM1* and *CaM4* could diversify their functions. Interestingly, potential miRNA target sites on *CaM1* differ from them on *CaM4* (Adai et al., 2005). Furthermore, various forms of splicing variants of *CaM1* are distinct from those of *CaM4*^2^. It would be of interest to figure out the molecular mechanism that renders the specificity of each *CaM* gene in plants.

## Author Contributions

JK and CD conceived the study and designed the experiments. CD and IL performed the experiments and analyzed the data with YL, HN, and JK. CD, YL, HN, and JK wrote the manuscript.

## Conflict of Interest Statement

The authors declare that the research was conducted in the absence of any commercial or financial relationships that could be construed as a potential conflict of interest.

## Footnotes

1 http://bbc.botany.utoronto.ca/efp/cgi-bin/efpWeb.cgi

2 http://www.arabidopsis.org

3 http://wmd3.weigelworld.org/cgi-bin/webapp.cgi

4 http://www.ncbi.nlm.nih.gov/

5 http://www.ebi.ac.uk/Tools/msa/clustalo/

## Supplementary Material

FIGURE S1 The expression pattern of *CaM* genes. **(A)** Expression of the *CaM* family genes in different tissues. Data were obtained from ePF-Browser (http://bbc.botany.utoronto.ca/efp/cgi-bin/efpWeb.cgi). Log2-transformed read counts without normalization were used in the computation. **(B)** Relative expression of *CaM1* compared to *CaM4* (http://bbc.botany.utoronto.ca/efp/cgi-bin/efpWeb.cgi). The result indicates the expression of *CaM1* and *CaM4* are almost identical in most of the tissues, except *CaM1* is highly expressed in pollen while *CaM4* highly expressed in dry seeds. **(C)** qRT-PCR analysis of *CaM1* and *CaM4* in different plant tissue. Expression levels of the genes were normalized to *Actin2*. Error bars represent means ± SEM of three independent experiments.

FIGURE S2 Overexpression of *CaM1-GFP* has no effect on seed size and mass. **(A)** Representative images of mature seeds of WT and *35S::CaM1-GFP* transgenic plants. **(B,C)** The relative seed size **(B)** and weight of 1,000 seeds **(C)** were measured in WT and *35S::CaM1-GFP* transgenic plants. Error bars represent means ± SEM (*n* = 5). Scale bars, 5 mm.

FIGURE S3 Functional redundancy between *CaM1* and *CaM4* in age-dependent leaf senescence. **(A,B)** qRT-PCR analysis of senescence related genes in *35S::CaM1-GFP* transgenic lines **(A)** and *35S::amiRNA-CaM1* plants **(B)**. Expression levels of the genes were normalized to *Actin2*. Error bars represent means ± SEM of three independent experiments. **(C)** Senescence phenotypes of WT and *cam4*. **(D)** Survival rates of WT and *cam4*. Plants with third and fourth rosette leaves showing complete yellowing were counted in WT (*n* = 4) and *cam4* (*n* = 8). In each experiment, 21–25 plants were analyzed. Statistical analysis was performed using heteroscedastic *t*-test (^∗∗^*p* < 0.01). Scale bars, 1 cm.

FIGURE S4 Protein sequence alignment of the calmodulin family. The EF-hand motifs of CaM1 were conserved across various homologs, including *Zea mays* (gi|162462264), *Glycine max* (gi|351721559), *Chlamydomonas reinhardtii* (gi|167411, gi|159490918), *Acyrthosiphon pisum* (gi|209870032), *Triticum aestivum* (gi|291246022), *B. napus* (gi|497992), *B. juncea* (gi|899058), *B. oleracea* (gi| 374922807), *Hevea brasiliensis* (gi|313767030), *Arabidopsis thaliana* (CaM2 to CaM7), and *Oryza sativa* (gi|17066588). Black lines indicate EF-hand motifs. Red asterisks indicate phosphorylation sites.

FIGURE S5 Phylogenetic analysis of the calmodulin family proteins.

## Abbreviations

ABA: abscisic acid
CaM1: calmodulin 1
DAB: diaminobenzidine
H_2_DCF-DA: dichlorofluorescein diacetate
H_2_O_2_: hydrogen peroxide
MeJA: methyl jasmonate
NBT: nitroblue tetrazolium
NO: nitric oxide
RbohF: reactive burst oxidase homolog F
RPK1: receptor-like potein kinase 1
SAG12: senescence-associated gene 12
