# Supplementary figures and images for: Calmodulin 1 Regulates Senescence and ABA Response in Arabidopsis
Source: Front Plant Sci. 2018 Jul 2;9:803. doi: 10.3389/fpls.2018.00803 (PMC6036150; doi:10.3389/fpls.2018.00803)

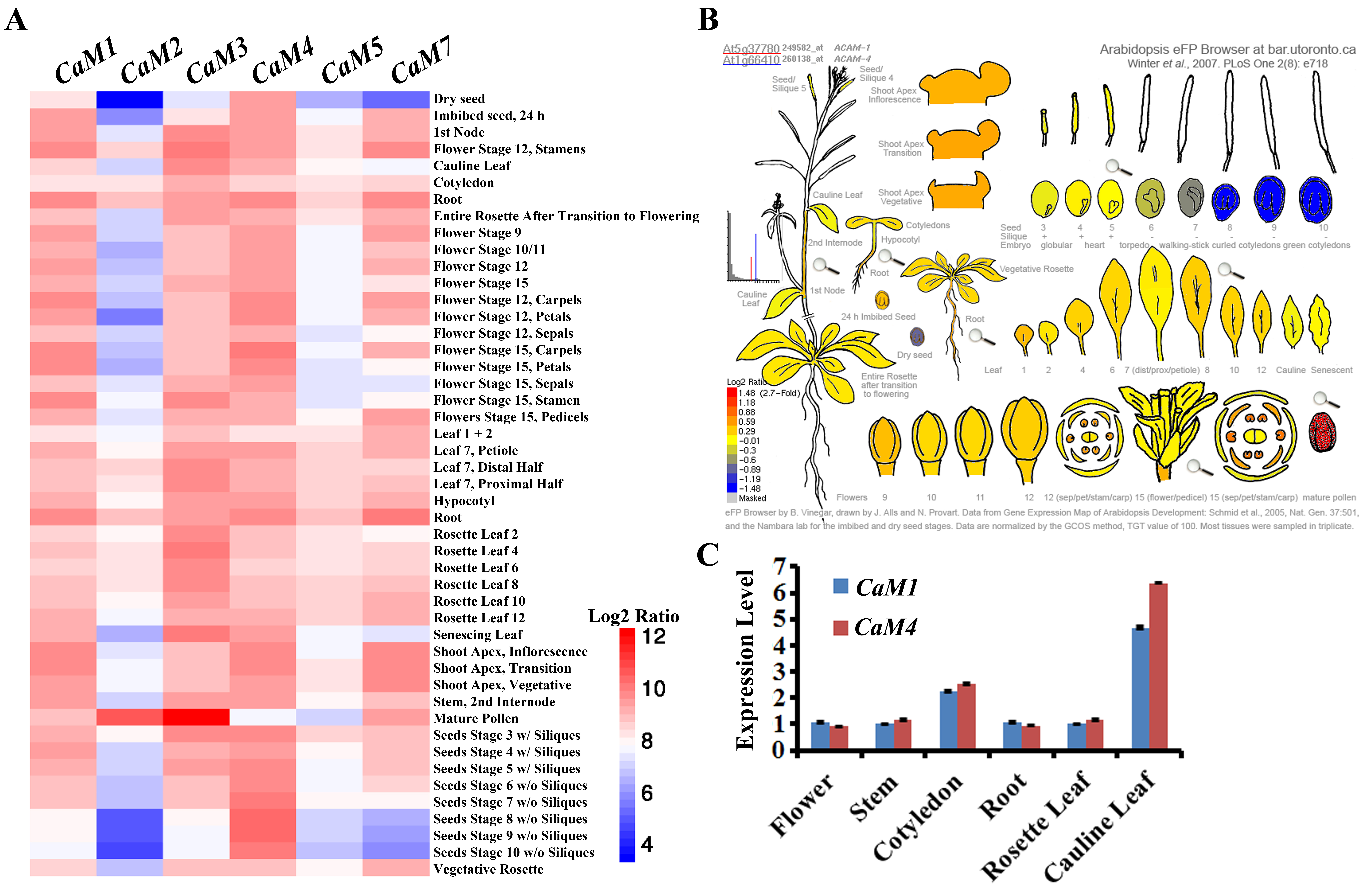

Supplement: FIGURE S1 — The expression pattern of CaM genes. (A) Expression of the CaM family genes in different tissues. Data were obtained from ePF-Browser (http://bbc.botany.utoronto.ca/efp/cgi-bin/efpWeb.cgi). Log2-transformed read counts without normalization were used in the computation. (B) Relative expression of CaM1 compared to CaM4 (http://bbc.botany.utoronto.ca/efp/cgi-bin/efpWeb.cgi). The result indicates the expression of CaM1 and CaM4 are almost identical in most of the tissues, except CaM1 is highly expressed in pollen while CaM4 highly expressed in dry seeds. (C) qRT-PCR analysis of CaM1 and CaM4 in different plant tissue. Expression levels of the genes were normalized to Actin2. Error bars represent means ± SEM of three independent experiments. [file Image_1.JPEG]

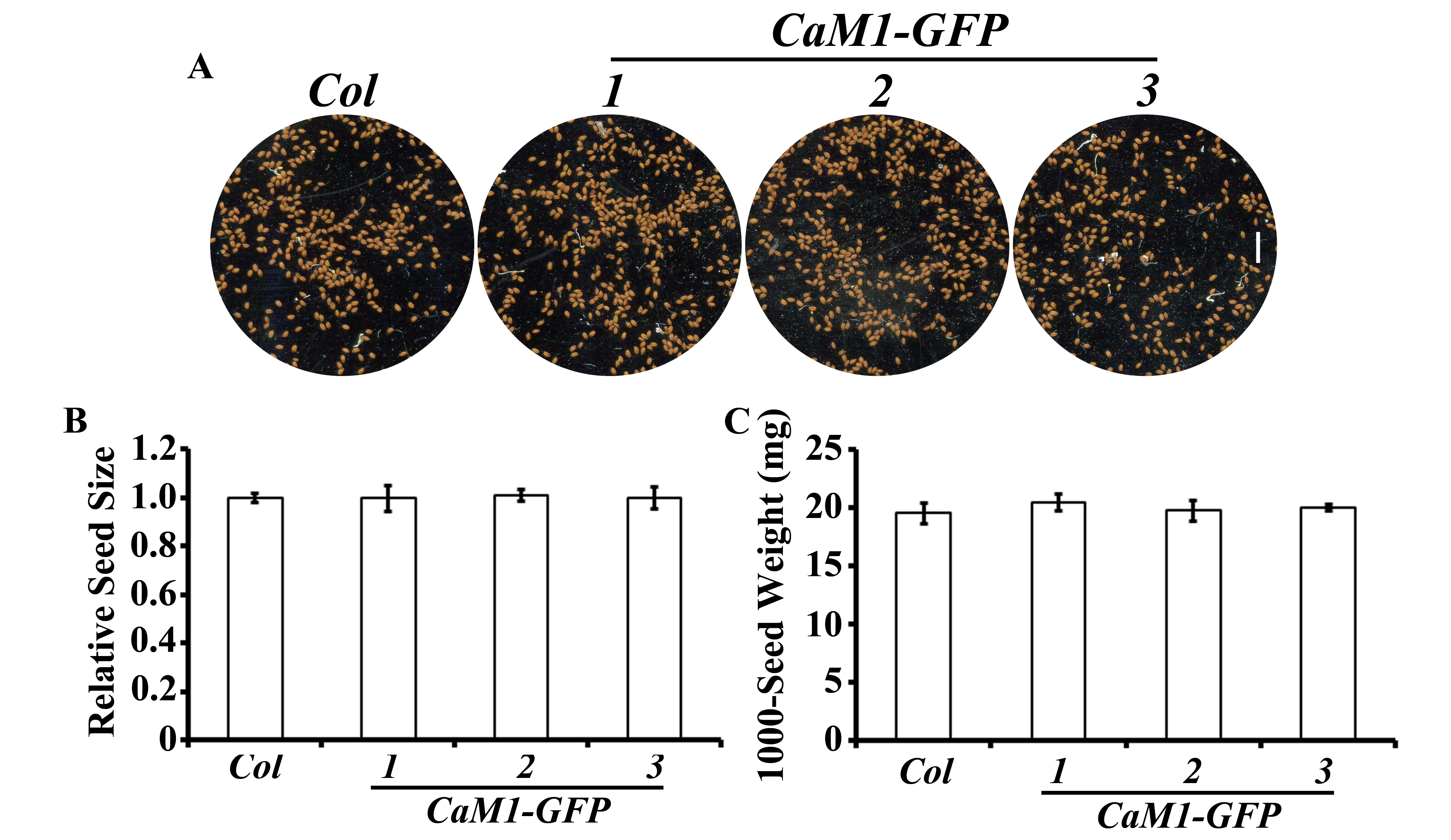

Supplement: FIGURE S2 — Overexpression of CaM1-GFP has no effect on seed size and mass. (A) Representative images of mature seeds of WT and 35S::CaM1-GFP transgenic plants. (B,C) The relative seed size (B) and weight of 1,000 seeds (C) were measured in WT and 35S::CaM1-GFP transgenic plants. Error bars represent means ± SEM (n = 5). Scale bars, 5 mm. [file Image_2.JPEG]

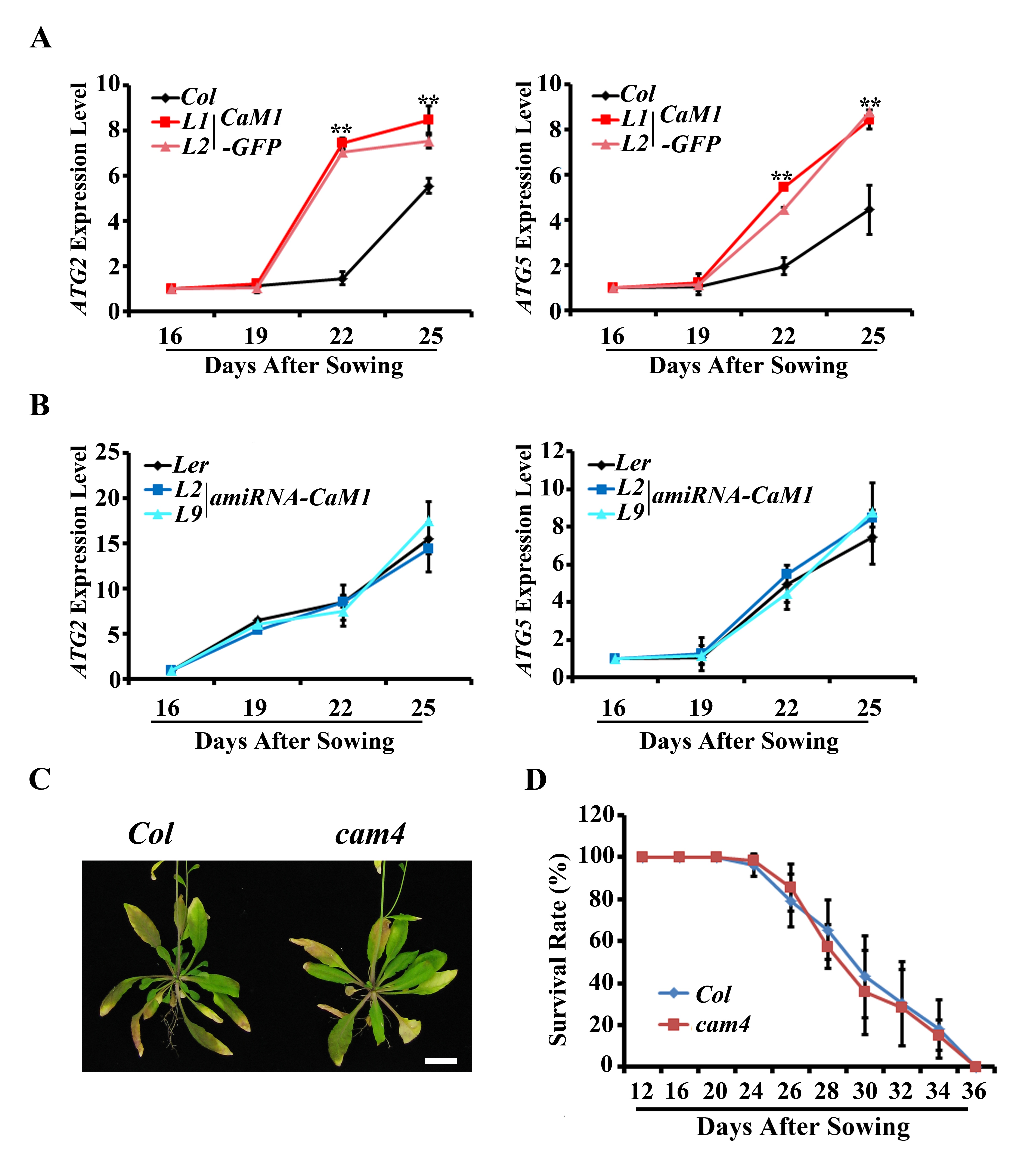

Supplement: FIGURE S3 — Functional redundancy between CaM1 and CaM4 in age-dependent leaf senescence. (A,B) qRT-PCR analysis of senescence related genes in 35S::CaM1-GFP transgenic lines (A) and 35S::amiRNA-CaM1 plants (B). Expression levels of the genes were normalized to Actin2. Error bars represent means ± SEM of three independent experiments. (C) Senescence phenotypes of WT and cam4. (D) Survival rates of WT and cam4. Plants with third and fourth rosette leaves showing complete yellowing were counted in WT (n = 4) and cam4 (n = 8). In each experiment, 21–25 plants were analyzed. Statistical analysis was performed using heteroscedastic t-test (∗∗p < 0.01). Scale bars, 1 cm. [file Image_3.JPEG]

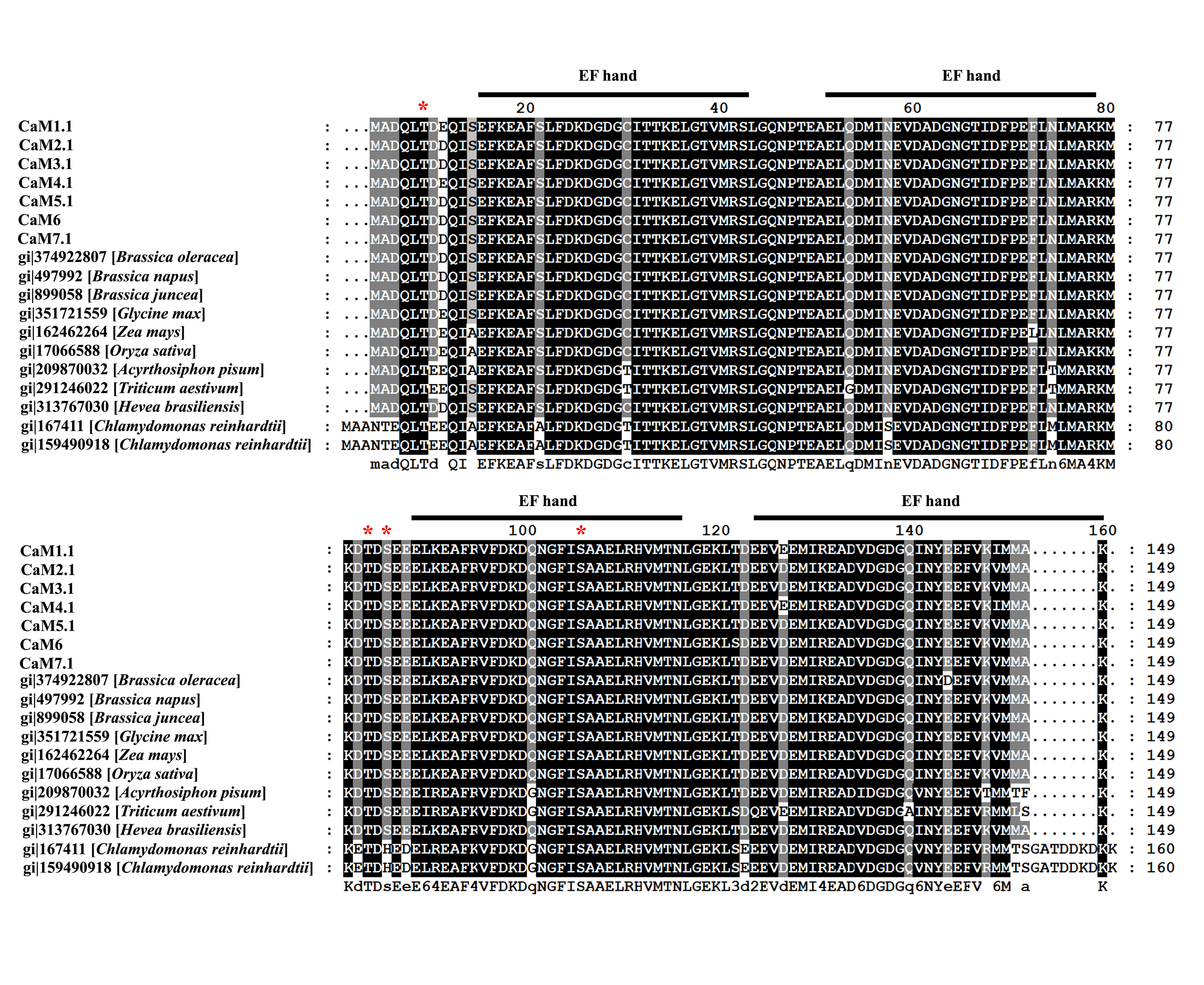

Supplement: FIGURE S4 — Protein sequence alignment of the calmodulin family. The EF-hand motifs of CaM1 were conserved across various homologs, including Zea mays (gi|162462264), Glycine max (gi|351721559), Chlamydomonas reinhardtii (gi|167411, gi|159490918), Acyrthosiphon pisum (gi|209870032), Triticum aestivum (gi|291246022), B. napus (gi|497992), B. juncea (gi|899058), B. oleracea (gi| 374922807), Hevea brasiliensis (gi|313767030), Arabidopsis thaliana (CaM2 to CaM7), and Oryza sativa (gi|17066588). Black lines indicate EF-hand motifs. Red asterisks indicate phosphorylation sites. [file Image_4.JPEG]

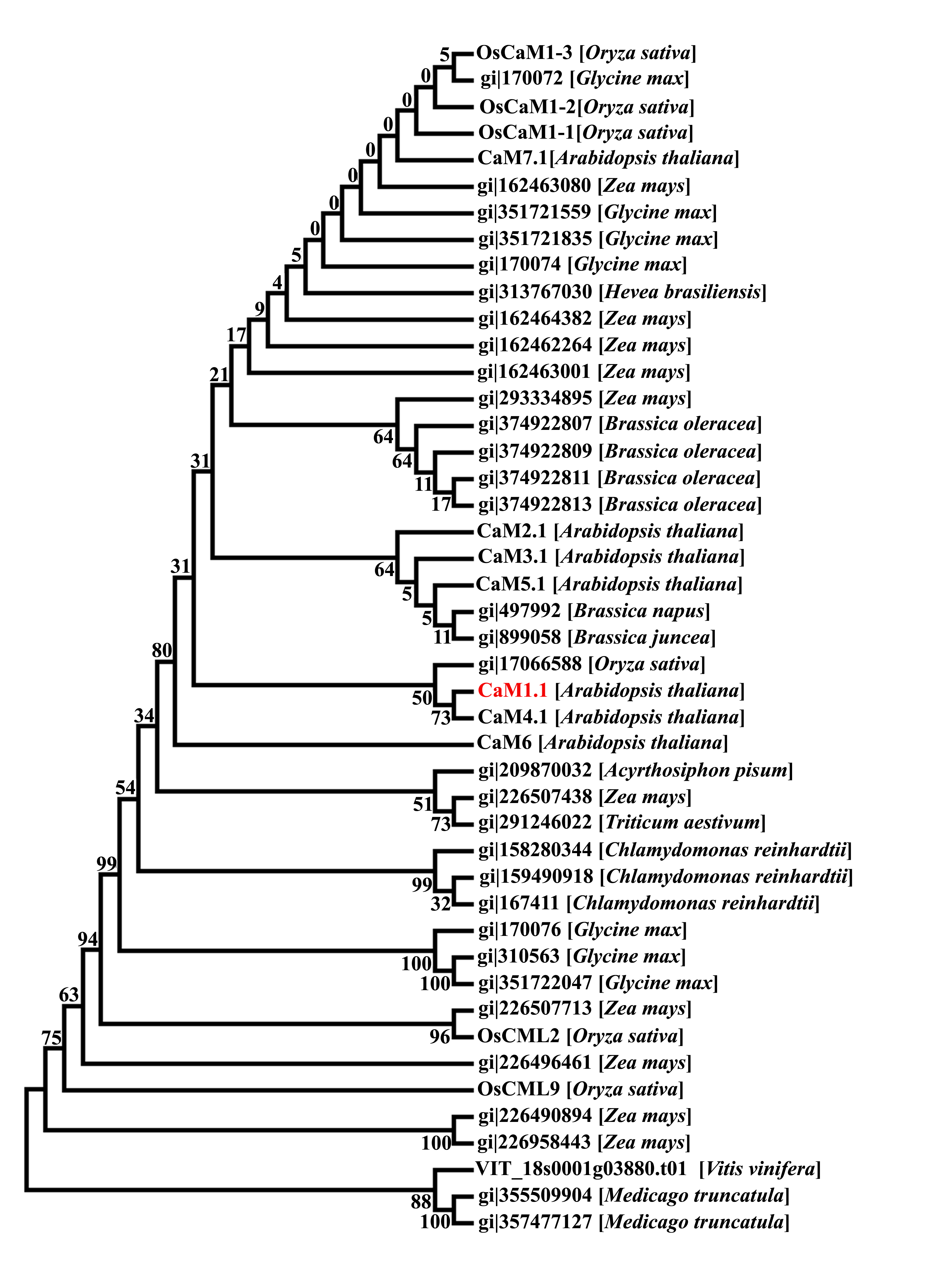

Supplement: FIGURE S5 — Phylogenetic analysis of the calmodulin family proteins. [file Image_5.JPEG]
